# Conservation benefit-sharing mechanisms and their effectiveness in the Greater Serengeti Ecosystem: local communities’ perspectives

**DOI:** 10.1007/s10531-023-02583-1

**Published:** 2023-04-06

**Authors:** Juma J. Kegamba, Kamaljit K. Sangha, Penelope A.S. Wurm, Stephen T. Garnett

**Affiliations:** 1 College of African Wildlife Management, Mweka, P.O. Box 3031, Moshi, Kilimanjaro Tanzania; 2 Research Institute for the Environment and Livelihoods, Charles Darwin University, Darwin, NT Australia

**Keywords:** Benefit-sharing mechanisms, Conservation, Greater Serengeti Ecosystem, Indigenous and local communities, Livelihood support, Social service provision

## Abstract

Benefit-sharing mechanisms have been instrumental in securing the support of local communities living on the edge of protected areas to implement protected area goals and enhance biodiversity conservation outcomes. Understanding the acceptability of the types of benefit provided among diverse communities is crucial for co-designing benefit-sharing approaches that accommodate local perspectives. Here, we used quasi-structured questionnaires and focus group discussions (FGD) to assess the acceptance of the types of benefit received by the communities in the Greater Serengeti Ecosystem (GSE) in Tanzania and the effectiveness of the benefits in securing community support for conservation reserves. We found that the categories of social service provision, livelihood support, and employment described all the benefits provided across conservation institutions operating in the GSE. However, the types of benefit within these categories varied significantly among conservation institutions, in terms of level and frequency of benefits received by communities. Overall, student scholarships were highly rated by respondents as the most satisfying benefit received. Respondents who were dissatisfied with the benefits received thought that the benefits did not compensate for the high costs arising from wildlife incursions onto their land. Communities’ acceptance of the benefits received varied greatly among villages, but only a small proportion of pooled respondents (22%) were willing to support the existence of a protected area without benefit. This study suggests that local people are willing to support conservation outcomes but require conservation institutions to give greater consideration to the costs incurred by communities, their livelihood needs, and access to natural resources or other benefits. We recommend that benefit-sharing be tailored to the local circumstances and cultures of people living close to protected areas, particularly communities expressing more negative views, to ensure adequate and appropriate compensation is provided.

**Supplementary Information:**

The online version contains supplementary material available at 10.1007/s10531-023-02583-1.

## Introduction

With over 30% of its land included within protected areas, Tanzania contributes more than most countries in the world to Aichi Target 11 in terms of the proportion of its land area protected for conservation purposes (WPA 2021; URT 2022a). One of the largest protected areas in Tanzania is the Greater Serengeti Ecosystem (GSE), which encompasses 25,000 km^2^ in the northern part of the country. With highly diverse wildlife and spectacular landscapes, the GSE is a leading global tourism destination and a major source of revenue for the country.

However, this success has come at a cost to local communities in and around the GSE (Kideghesho 2008; Mwakaje et al. 2013). During the establishment of the protected areas that form the GSE, traditional communities were expelled from their traditional lands, with little or no consultation or compensation (Songorwa 1999). Such expulsions continue with ongoing evictions of Maasai pastoralists because their increased population is perceived as a threat to the quality of one of the leading tourist attractions in the country (Al Jazeera 2022; Mittal 2022). The livelihoods of Indigenous and other local people living in and around the GSE have also been significantly compromised because wildlife from the GSE causes crop damage, injures and kills people and livestock, and competes for pasture and other natural resources (Kideghesho and Mtoni 2008; Mwakaje et al. 2013; Melubo and Lovelock 2019). Overall, any benefits received from conservation by the local communities in and around the GSE have come at a substantial cost (Mwakatobe et al. 2014; Eustace et al. 2018).

The sharing of benefits derived from biodiversity conservation with relevant Indigenous or other local peoples is among the five strategic goals of the Convention on Biological Diversity (CBD 2010). Under this goal, local people with knowledge of, and interests in, lands under conservation tenure have the right to receive a share of the benefits derived from their traditional lands (UN General Assembly 2007; UN Secretariate CBD 2011). Sharing the benefits generated from the protected areas aims not only to compensate communities for losses resulting from their proximity to these areas but also to improve their attitudes toward conservation (Archabald and Naughton-Treves 2001; Salafsky et al. 2001; Parker et al. 2022).

In Sub-Saharan Africa, such benefits are provided to communities by distributing the fees paid by visiting tourists (Campbell and Shackleton 2001; Ahebwa et al. 2012). These arrangements have three forms: (i) a proportion of the cash revenue received by the national government is distributed to communities for development projects (Schroeder 2008; Vedeld et al. 2012; Mariki 2013); (ii) private companies share profits derived from businesses operating on community lands, such as hunting (Lindsey et al. 2007; Saarinen et al. 2009; Ochieng 2011) and (iii) some public and private entities work collaboratively and share profits with the communities under a memorandum of understanding to operate businesses (Spenceley et al. 2019).

In Tanzania as a whole, a majority of local communities living near conserved areas receive some benefits provided by conservation institutions (Kegamba et al. 2022). Such benefit-sharing programs have evolved over time. For example, in 1988, the Community Conservation Services (CCS) program provided support for the construction of schools, health centres, and water wells in the nearby villages (Kaaya and Chapman 2017). In 2006, the first Wildlife Management Area (WMA) was gazetted, allowing local people to manage and use wildlife resources in a conserved area outside the more strictly conserved game reserves and national parks (URT 2002a; Schmitt 2010). In 2009, the Frankfurt Zoological Society (FZS) helped establish the so-called Community Micro-credit Program or Community Conservation Bank (COCOBA) in the GSE (Sulle 2012; Kaaya and Chapman 2017).

There has also been some recognition that living near a protected area can incur costs. There is compensation for the loss. While compensation for loss does not constitute a benefit as such, it is consolation for loss. The creation of consolation scheme to compensate for losses from dangerous animals has the same objective of increasing the willingness of communities to accept protected areas. To this end the Ministry of Natural Resources and Tourism introduced a consolation scheme in 2011 for communities affected by seven species of dangerous wildlife i.e. Wildlife Conservation (Dangerous Animals Damage Consolation) Regulation (URT 2011). This regulation applies to all protected areas in Tanzania. Under the regulation, claimants can receive TZS 1 million (USD 432) for loss of a human life, of 20 cattle or of 40 sheep/goats or up to TZS 100,000 (USD 43) per acre of lost cropland up to 5 acres. This regulation applies if the claimant’s loss happened more than 0.5 km from a protected area boundary. This consolation does not apply to losses within this 5 km buffer. Conditions for compensation are stringent and the penalties for false claims are high.

Overall, the acceptance of conservation benefits is strongly linked to the history of engagement between communities and conservation institutions (Kegamba et al. 2022). For example, the benefits from the WMAs are considered more favourably by the local communities due to their effective engagement than those provided by the national parks (Kegamba et al. 2022). As a result, the WMAs have tended to engender strong community support because communities are involved in the decision-making process and conservation benefits contribute to improving local livelihoods (Campbell and Shackleton 2001; Nelson et al. 2007). However, there have recently been conflicts over the allocation and use of benefits from WMAs, meaning some communities are now considering withdrawing their cooperation (Bluwstein et al. 2016; Moyo et al. 2017; Kicheleri et al. 2018, 2021). Such dissatisfaction with benefit sharing, where local perspectives and priorities have not been accommodated, has been documented elsewhere in Selous Game Reserve in Tanzania (Gillingham and Lee 1999), as well as in Zimbabwe (Shereni and Saarinen 2021; Parker et al. 2022) and India (Arjunan et al. 2006).

Key challenges for improving the acceptability of benefit-sharing mechanisms for communities around conserved areas generally and the GSE in particular, are the diversity of communities and the types of benefit being provided. Communities around the GSE include hunter-gatherer communities with no fixed village base and no livestock, mobile pastoralists who focus on raising livestock, and agro-pastoralists with livelihoods based on both livestock and cropping. The differences in culture, livelihood strategies and history (Kaltenborn et al. 2008; Kideghesho 2008; Estes et al. 2012; Sekar et al. 2014) are likely to have profound effects on local people’s acceptance and perceptions of the benefits received from different conservation institutions involved with the GSE. Throughout Tanzania, the types of benefit also vary greatly, from direct cash to a range of cash-free social benefits (Kegamba et al. 2022), but there is little contemporary knowledge of which form of benefit provided is most appreciated by the communities. This study aims to fill this knowledge gap by investigating the acceptance of benefits received by three community types with differing livelihood strategies (hunters and gatherers, pastoralists and agro-pastoralists), who are affected by conservation in the GSE. Specifically, we assessed: (i) the types of benefit received by the communities from the GSE protected areas, (ii) acceptance of those benefits by the communities and (iii) effectiveness of the benefits in securing community support for conservation in the GSE. This research will inform the development of better benefit-sharing approaches in consultation with local communities that will deliver improved community livelihoods and a genuine commitment to the goals of the conserved areas in the GSE and elsewhere.

## Study area and methods

### Institutional context

**Fig. 1:**
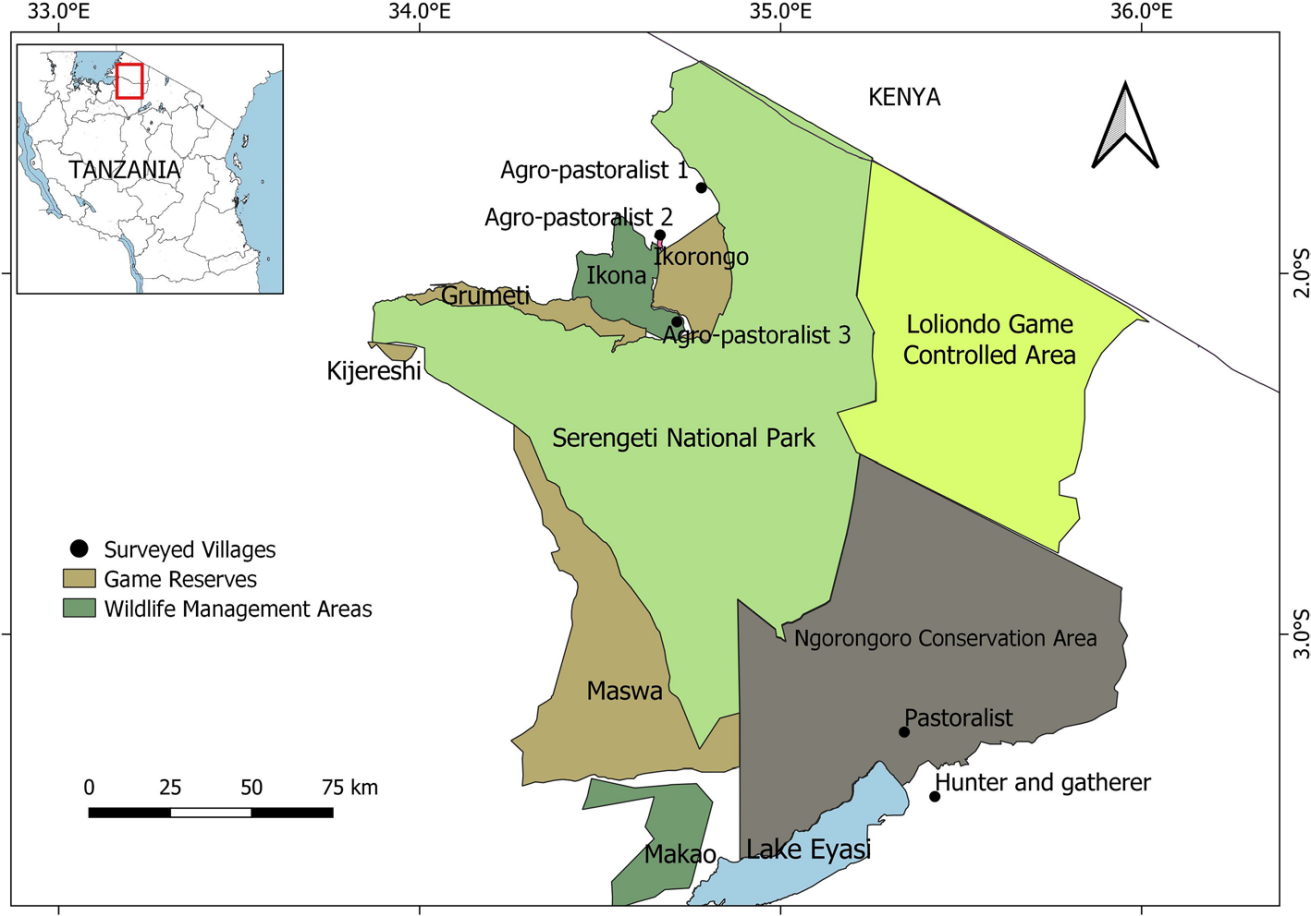
Protected areas in the Greater Serengeti Ecosystem (GSE) of Tanzania and the location of the five villages surveyed for this study (indicated by labelled black dots)

The study was conducted with communities living in and around protected areas in the GSE, covering an area of 25,000 km^2^ in north-western Tanzania and south-west Kenya (Fig. 1). Much of the region, which is exceptionally rich in wildlife, has been incorporated into protected areas. The protected areas in the GSE are managed by five different government institutions (Table 1).

Each of the five conservation institutions in the GSE has different management objectives and benefit-sharing schemes for the local communities (Mfunda et al. 2012; Mwakaje et al. 2013; Kisingo et al. 2016). The Serengeti National Park excludes any human activities not related to conservation while the four game reserves allow game hunting by tourists. Although multiple benefits are provided to people living in the Ngorongoro Conservation Area, farming is strictly prohibited. Local people have more rights within the two WMAs than under the other conservation tenures. The two WMAs in the GSE were established to promote both the long-term survival of wildlife and the economic development of the local people, with communities directly involved in managing and using the wildlife resources (URT 2002a).

Tourism companies that generate profits in the GSE protected areas also provide benefits to communities. The four companies operating in the study area were the Bush Top Tour Company, Mbuzi Mawe Tourist Camp, Thomson Safari Company and Tanzania 2000 Adventure Company. Communities in the study area were also supported by two not-for-profit environmental organizations, the Grumeti Fund Organization and the World Wildlife Fund (WWF), and by one professional association, the Frankfurt Zoological Society (FZS).

**Table 1:** Conservation institutions managing the Greater Serengeti Ecosystem, Tanzania, and their key characteristics

Name protected area	Tanzanian managing authority	IUCN category	Area (km^2^)	
Serengeti National Park	Tanzania National Parks (TANAPA)	II (National Park), conservation and non-consumptive tourism	14,700	
Ngorongoro Conservation Area	Ngorongoro Conservation Area Authority (NCAA)	III (Natural Monument) and V (Protected Landscape), protection of specific natural monument (Crater), and conservation of biodiversity, cultural and scenic values	8,200	
Ikorongo and Grumeti Game Reserves	Tanzania Wildlife Management Authority (TAWA)	VI Managed Resource Protected Areas—consumptive and non-consumptive tourism	3,797	
Maswa Game Reserve	Tanzania Wildlife Management Authority (TAWA)	VI Managed Resource Protected Areas—consumptive and non-consumptive tourism	2,765	
Kijereshi Game Reserve	Tanzania Wildlife Management Authority (TAWA)	VI Managed Resource Protected Areas—consumptive and non-consumptive tourism	66	
Loliondo Game Controlled Area	Tanzania Wildlife Management Authority (TAWA)	VI Managed Resource Protected Areas	4,200	
Ikona Wildlife Management Area	Wildlife Management Area (WMA)	VI Managed Resource Protected Areas	242	
Makao Wildlife Management Area	Wildlife Management Area (WMA)	VI Managed Resource Protected Areas	769	

Source: Kisingo et al. (2016)

### Local community context

The local communities in and around the GSE (Fig. 1) vary in terms of their history, land-use, culture, livelihood strategies, and land tenure arrangements (Kaltenborn et al. 2008; Estes et al. 2012). All land occupied by local communities is owned by the Government of Tanzania which, outside the protected areas, allows customary rights of occupancy under the Village Land Act (African Natural Resources Centre, undated), although tenure rights are complex (Locher 2016).

The smallest community participating in the research were the hunter-gatherer Hadzabe communities (also called Hadza, Tindiga or Watindiga), believed to have occupied the eastern part of the GSE over 10,000 years ago (Marlowe 2002; Kideghesho 2008). In the late 20th century, in response to pressure from the agro-pastoralist people in the western Serengeti, and pastoralists (Maasai and Datoga) from the north and east of the GSE, the Hadzabe people moved to the south of the GSE to an area near Lake Eyasi (Kideghesho 2008) (Fig. 1). The Hadzabe community in Tanzania is one of the few societies in the world that still practices hunting and gathering as their main livelihood strategy (Marlowe 2002).

The second community grouping is the Maasai and Datoga pastoralist communities, which migrated from South Sudan to Kenya in the 15th century and later settled in northern Tanzania (Nilsson 2016). During the time of arrival of Europeans in the 17th century, some Maasai communities were living in the Serengeti plains (Århem 1985) while the Datoga communities primarily lived on the Ngorongoro crater and its highlands (Nilsson 2016). The Maasai people were relocated from the Serengeti plains to the Ngorongoro Conservation Area in the 1950s by the British Colonial Government as a consequence of the establishment of the Serengeti National Park (Århem 1985; Sirima and Backman 2013). In the early 1990s, the Maasai people then displaced the Datoga people who moved from the Ngorongoro highlands to the rift valley basin near Lake Eyasi (Nilsson 2016). There they established the villages of Olpiro and Masamburai inside the NCA (Olpiro Village Chairperson; personal communication, 2022). Livestock keeping, mostly cattle, remains the primary livelihood strategy for both Datoga and Maasai people living inside the Ngorongoro Conservation Area, where agriculture is prohibited (Diarz et al. 2020).

The third and largest community grouping is the agro-pastoralist communities who live along the western border of the GSE and identify as Kurya, Ikoma, Sukuma, Isenye, Ngoreme, Zanaki, Jita and Luo (Kideghesho et al. 2007). Some of these tribes, such as the Ikoma, have long had cultural connections to the GSE, from which they obtained bush meat before being relocated to the periphery of the protected areas during their establishment (Kideghesho et al. 2007; Kideghesho 2008; Sangha et al. 2018). These agro-pastoralist communities combine livestock keeping – mainly cattle, sheep and goats – with manual cultivation of maize, finger millet, beans, cassava, sorghum and tobacco on small farms of 0.9 to 3 ha (Kideghesho et al. 2007; Estes et al. 2012).

### Data collection

#### Sampling

Information on local people’s perspectives on the types of benefit received, and effectiveness of these benefits in securing community support for conservation in the GSE, was collected from five villages/communities through focus groups and individual interviews. For data collection, stratified and cluster sampling techniques (Kijazi and Kant 2011) were adopted. First, we stratified communities as those geographically located within or beside a protected area in the GSE managed by a conservation institution (Zafra-Calvo et al. 2017). Second, we categorised communities as belonging to one of the following groups based on their livelihood strategies: hunter and gatherer peoples, pastoralist, and agro-pastoralist communities. There is only one hunter-gatherer group living near the GSE, the Hadzabe people. Of the two pastoralist communities, we selected the Datoga people; an intention to interview Maasai community was thwarted by the political tension over the proposed eviction of Maasai people from the Ngorongoro Conservation Area (Mittal 2022). Third, we used a non-probability sampling technique to select “representative sample villages” among many villages constituting the local community stratum (Zafra-Calvo et al. 2017), eventually selecting villages belonging to Ikoma and Kurya people. Agro-pastoralist village 1 was located next to Serengeti National Park away from the main road, Agro-pastoralist village 2 was on the main road into the park at the junction of Ikorongo Game Reserve and Ikona Wildlife Management Area and Agro-pastoralist village 3 was close to the main entrance and administrative centre of Serengeti National Park as well as close to Ikorongo and Grumeti game reserves, and Ikona Wildlife Management Area.

While “village” is an appropriate term for describing clusters of households sampled from pastoral and agro-pastoral communities, we also used this term to encompass the community of Hadzabe people who did not live in established villages but moved frequently between them. The Hadzabe people were living in Division Eyasi when interviewed (March 2022), the Datoga pastoralists were from Olpiro Village and the agro-pastoralists communities lived in the villages of Bisarara, Park Nyigoti and Robanda. Before data collection, we obtained a research permit from Tanzania’s Commission for Science and Technology (COSTECH) No. 2021-476-NA-2021-170 and an introductory letter from the respective district councils, Serengeti and Karatu. Within each village, households were selected based on their accessibility and availability of household heads for interviews. The research was conducted under Charles Darwin University Human Research Ethics Committee Permit number H21058.

#### Household surveys

We surveyed 296 households, i.e. 13.2% of the total households (~ 2,245) in the study areas. This included 60 hunter/gatherers (~ 60%), 86 in a pastoralist village (25%) and 50 from each of three agro-pastoralist communities (9%, 17% and 25% of all households in each village). With each household randomly selected, we undertook a quasi-structured questionnaire, defining a household as a group of people living together, mostly with a single person who self-identified as its head. If the head of the household was not available, we chose the nearest household instead. The interviews began by seeking verbal approval of respondents after they were informed of their right to withdraw from the interview at any stage, and that whatever had been recorded would be redacted from the research records, if they did decide to withdraw. At each household we asked respondents the following questions: (i) What types of benefit did you receive as a result of living in or near a protected area? and (ii) Did you find the benefits received acceptable? (answers recorded on a Likert scale: (1) Strongly reject; (2) Reject; (3) Neither reject nor accept; (4) Accept; (5) Strongly accept). We also asked respondents to name the benefit with which they were most satisfied. We then asked respondents a mix of closed and open-ended questions about their perspectives on the effectiveness of the benefits received in securing community support for the conservation of protected areas.

Interviews were largely conducted in the Kiswahili language, a national language in Tanzania in which the lead author is fluent, with a translator employed to conduct discussions in the local language when required. The interviews were recorded, transcribed, and translated into English for analysis by the lead author.

#### Focus group discussion

To cross-reference survey results, we conducted a Focus Group Discussion (FGD) with a small group of participants (~ 10) in each of the villages visited for household questionnaire surveys. Two FGDs were held in each village for people aged over 20 years, one for men and one for women, making a total of 12 in all. The discussion was guided by a checklist of questions drawn from the research objectives to stimulate participants’ contributions. Specifically, we focused on the types of benefit received from conservation institutions, how often the communities received benefits, and who within the village benefited. We tabled the benefits mentioned during the questionnaire survey for confirmation in each village’s FGD. The first author facilitated and ensured that participation and the discussion proceeded until participants ran out of questions/queries on each topic (Newing 2011, 104). As with the interviews, FGDs were recorded, transcribed, and translated into English for analysis by the lead author.

### Data analysis

#### Qualitative analysis

Categories of benefits were based on key research questions (Patton 2002; Ritchie and Spencer 2002) and grouped based on a recent review of benefit-sharing mechanisms in the region by Kegamba et al. (2022) as *social services provision*, *livelihood support* benefits, and *employment* of the local people.

*Social services provision* included all social benefits received such as an investment in schools (in the form of classrooms, teachers’ houses or offices, student desks, dormitories, scholarships or books), health centers, ambulance services, village office buildings and funds received for village development projects. *Livelihood support* included benefits which fulfill locals’ necessities of life such as food, water for domestic use or livestock, vaccination for livestock, loans, health insurance, training (in, for example, materials for crafts, credits and production opportunities such as beekeeping), or access to non-timber forest products (NTFP). The *Employment* category included any employment received from the protected area, regardless of whether this included pensionable terms (permanent) or non-pensionable terms (temporary).

#### Quantitative analysis

Mean household acceptability for the different types of benefit was derived from the Likert scores in each village. We then employed binomial Generalized Linear Models (GLM) using R 3.6.2 (R Core Team 2019) to assess the influence of demographic variables (livelihood strategy, village, sub-village, gender, age and education level) upon a binary agreement response variable. The models addressed the following research questions on whether the communities.

1. viewed the benefit received as effective in encouraging them to support protected areas in the GSE;
2. viewed the benefit received as helpful in reducing illegal activities in the GSE;
3. are willing to support the existence of protected areas in the GSE without receiving any benefit.

Clustering by sub-village was explored by fitting a random intercept for sub-villages and performing a mixed-effect GLMM (R package lme4). This did not improve the model fit and was not followed further (data not shown). AICc was used for model selection. Model validation was performed using the DHARMa R package (Hartig 2020). Model residuals were checked for lack of overdispersion and lack of patterns across predictors and fitted values. Multivariable models were also checked for lack of collinearity. The R package lsmeans was used to calculate the average estimated probabilities and 95% CI based on the binomial model outputs.

## Results

### Respondent demographic characteristics

About 40% of respondents from the households were female, and the rest were male, within the age groups of 18–35 years (30%), 36–50 years (45%), and over 50 years (25%). Most household respondents (65%) had some formal education (primary, secondary or postsecondary) but 35% had not attended school. A large majority (85%) of respondents were born in the villages surveyed.

### Types of benefit received by the communities

The reported benefits received by each surveyed village varied in type, magnitude and frequency (Table 2). For example, the Pastoralist village located inside Ngorongoro Conservation Area received several benefits whereas Agro-pastoralist 1 bordering SENAPA received very few.

**Table 2:** Types of benefit received by local communities in the surveyed villages from in and around the GSE. Note that respondents were not always able to provide the timeframes and dates for the receipt of benefits

Villages/community surveyed	Name of organization or institution providing the benefits	Categories of benefit received		
		Social services received	Livelihood supported	Employment type received
Hunter and gatherer	NCAA^1^	Contributed to construction of primary school building, dormitory for the Hadzabe children and school toilet	Allowed to hunt wild animals (except national game and endangered species) for free and for subsistence only, allowed to collect NTFPs for free and provided maize grain at half market price every three months	Temporary casual labour occasionally available
Pastoralist	NCAA	Constructed two primary school classrooms and one teachers’ office, constructed four teachers’ houses, provided 50 students desks, constructed village office, provided 16 scholarships for children from secondary school to colleges every year since 2008, and constructed a cattle trough in the village, NCAA ambulance was made available for rapid transport to hospital anywhere in the country with NCAA paying half medication costs.	Allowed to collect NTFPs for free, provided food (maize grain) at half local market price, constructed water system for domestic use in the village, received nutritional food for primary school kids, received health insurance for 52 people from poor families, contributed beehives to beekeeping project groups, received free livestock vaccination (except East Coast Fever), received 35 Million TZS loan for entrepreneurship groups (20 groups). Received compensation for death or injury of people from dangerous wildlife while within their village	Temporary casual labour occasionally available
Agro-pastoralist 1	SENAPA^2^	Constructed four classrooms and provided student desks	None provided	None provided
	Bush Top Tour Company (tourism business) investing in SENAPA	Provided 160 student desks and constructed water harvesting tank for primary school	None provided	Temporary casual labour
	Frankfurt Zoological Society (not for profit) working with SENAPA	None provided	Helped establish two COCOBA^3^ groups and provided 46 beehives for bee keeping projects	None provided
	MNRT^7^	None provided	Received compensation for death or injury of people, stock or crops from dangerous wildlife while within their village	None provided
Agro-pastoralist 2	Grumeti Fund Organization (not for profit) investing in Ikorongo & Grumeti game reserves and Ikona WMA^4^	Constructed water harvesting tank for primary school, sponsored over 30 students since 2004 to attend schools and colleges in Tanzania, contributed to the construction of village health center	Constructed one water pump for the villagers	Temporary casual labour and few permanent employments available
	SENAPA	Constructed two primary school classrooms and one teachers’ office	Constructed one water dam for livestock in the village	Temporary casual labour occasionally available
	Mbuzi Mawe Tourist Camp (tourism business) investing in SENAPA	Constructed primary school kitchen and donated school uniform to best performing pupils	None provided	Temporary casual labour occasionally available
	WWF^5^	None provided	Facilitated establishment of three beekeeping project groups, provided 40 beehives	None provided
	Ikona WMA	Provided dividend of 611,000 USD (1,423 million TZS) from 2012 to 31st January, 2022 (revenue for village development projects)	Revenue used to purchase food for poor families during extreme hunger caused by prolonged drought	Temporary casual labour and permanent employment available
	MNRT^7^	None provided	Received compensation for death or injury of people, stock or crops from dangerous wildlife while within their village	None provided
Agro-pastoralist 3	SENAPA	Constructed village health center, donated 80 beds and mattresses for village boarding school	Constructed three water dams for livestock	Temporary casual labour available in tourist camps
	Grumeti Fund Organization (not for profit) investing in Ikororongo & Grumeti game reserves & Ikona WMA	Constructed water harvesting tank in village health center, sponsored over 30 students since 2004 to schools and colleges in Tanzania	12 Entrepreneurship groups from the community have entered memorandum of understanding with Grumeti Fund Organization for a tourist campsite investment on their land, receiving 90,000 USD (200 million TZS) per year	Temporary casual labour and few permanent employments available
	Thomson Safari Company (tourism business) investing in Ikona WMA	Constructed seven teachers’ houses, secondary school student dormitory, two school water pumps and primary school kitchen and donated books for students	Not applicable	Temporary casual labour and permanent employments were available before COVID-19 outbreak.
	Tanzania 2000 Adventure Company (tourism business) investing in Ikona WMA	Constructed one teacher’s house for primary school	Provided food for primary school kids for 2 years before COVID-19 outbreak	Temporary casual labour and permanent employment were available before COVID-19 outbreak.
	Ikona WMA	Provided dividend of 600,000 USD (1,422 million TZS) from 2012 to 2022 (revenue for village development projects)	Used annual received revenue to purchase food for poor families during extreme hunger caused by long drought	Temporary casual labour and permanent employment
	MNRT	None provided	Received compensation for death or injury of people, stock or crops from dangerous wildlife while within their village	None provided

*NCAA* Ngorongoro Conservation Area; *SENAPA* Serengeti National Park; *COCOBA* Community Conservation Bank; *WMA* Wildlife Management Area; *WWF* World Wide Fund for Nature; *NTFPs* Non-Timber Forest Products; *MNRT* inistry of Natural Resources and Tourism

### Acceptability of benefits

**Fig. 2:**
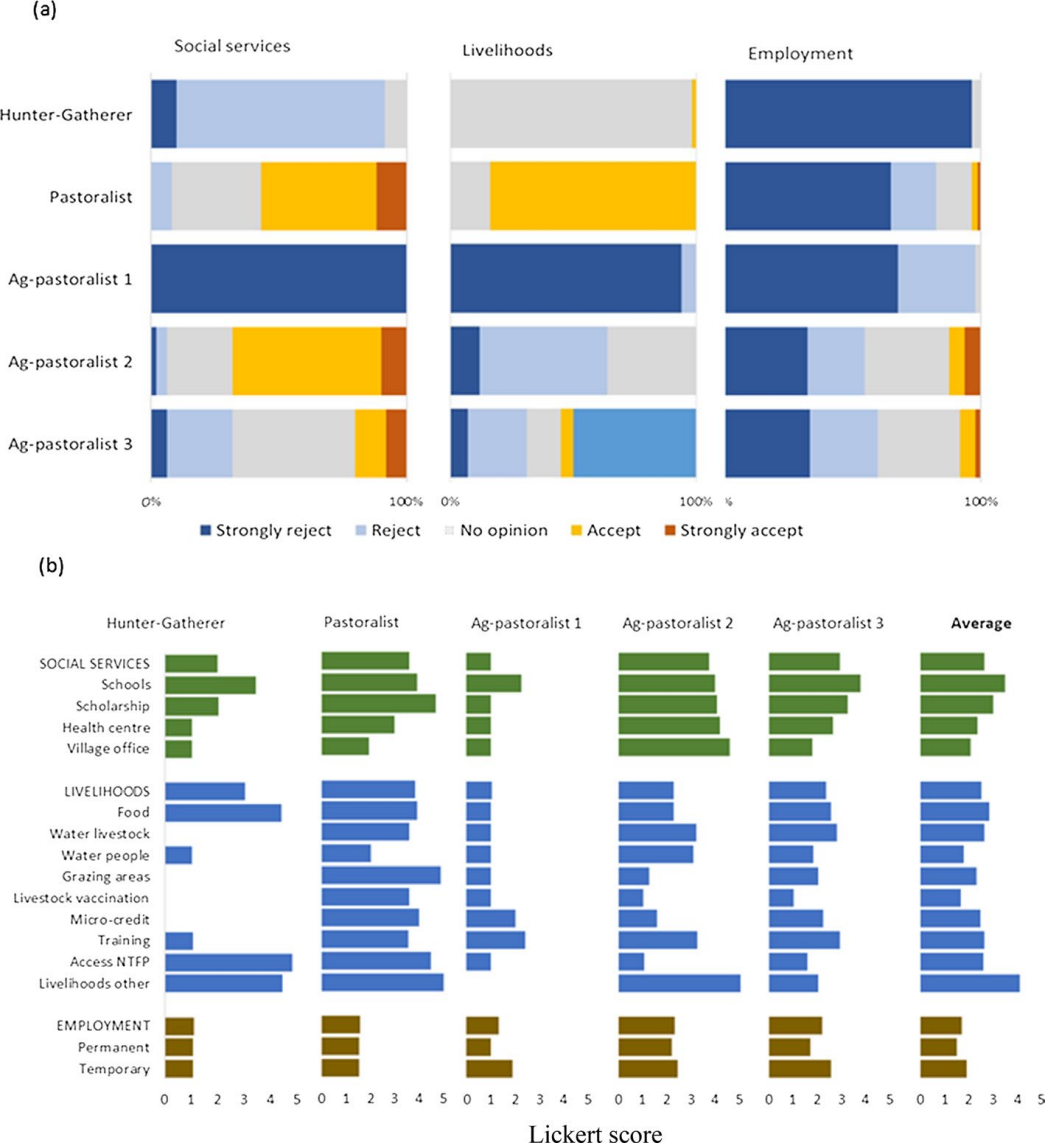
Acceptability of benefits received from conservation institutions in GSE as ranked by the respondents from the surveyed community per **a** categories of benefits  **b** individual benefits

Across all communities, the average acceptance level (Likert score) across all benefits was 2.2 (out of a maximum of 5). Social service benefits were ranked the highest (mean score of 2.8), followed by livelihood benefits (mean score of 2.4) and the least was employment (mean score of 1.6) for the pooled data (Fig. 2). While this suggests that most respondents were dissatisfied with the benefits provided, there were substantial differences among communities and within community groups (Fig. 2). Respondents from the Pastoralist village scored 4.0 for both social services and livelihood benefits while respondents from Agro-pastoralist 1 village had a mean score of 1 across all three types of benefit. Reasons given for the scores also varied greatly across the villages surveyed (Table 3).

**Table 3:** Main reasons given by the respondents for accepting or rejecting benefits provided by the institutions and organizations associated with the GSE

Village	Main reason why benefits considered acceptable	Main reasons why benefits considered unacceptable
Hunter and gatherer	Free access to NTFPs and hunting in Ngorongoro Conservation Area	Most hunter and gatherer people had been re-located to marginalised lands in Division Eyasi with little wildlife available for hunting. Also, their lands have been invaded by other communities and the government assistance to this community is inadequate.
Pastoralist	Received many benefits from NCAA, the most acceptable included most social and livelihood benefits	Farming restricted and ongoing fear of relocation from Ngorongoro Conservation Area
Agro-pastoralist 1	Two Community Conservation Bank (COCOBA) groups (30 people) established by Frankfurt Zoological Society are doing well and FZS are also involved in beekeeping projects	Cost incurred from wildlife including crop damage (mainly by elephants), livestock depredation (mainly hyenas, lions and leopards), attacks on humans (mainly elephants). Benefits inadequate and infrequent (e.g. SENAPA built school and provided desks in 1995). Long-standing boundary conflict between SENAPA and village
Agro-pastoralist 2	Received and appreciated a range of social and livelihood benefits from SENAPA, Ikona WMA, investors in SENAPA, Ikorongo & Grumeti game reserves. Well-constructed village office using WMA funds	Cost incurred from wildlife including crop damage (mainly by elephants), livestock depredation (mainly hyenas, lions and leopards), attacks on humans (mainly elephants). Long-running boundary conflict between the village and Ikorongo Game Reserve. Benefits too small and not directed to household needs
Agro-pastoralist 3	Accepted a range of social and livelihood benefits from SENAPA, Ikona WMA, investors in SENAPA, Ikorongo & Grumeti game reserves. In addition, some villagers have invested in conservation on their land, in partnership with significant investors	Cost incurred from wildlife including crop damage (mainly by elephants), livestock depredation (mainly hyenas, lions and leopards), attacks on humans (mainly elephants). Benefits too small and not directed to household needs

**Fig. 3:**
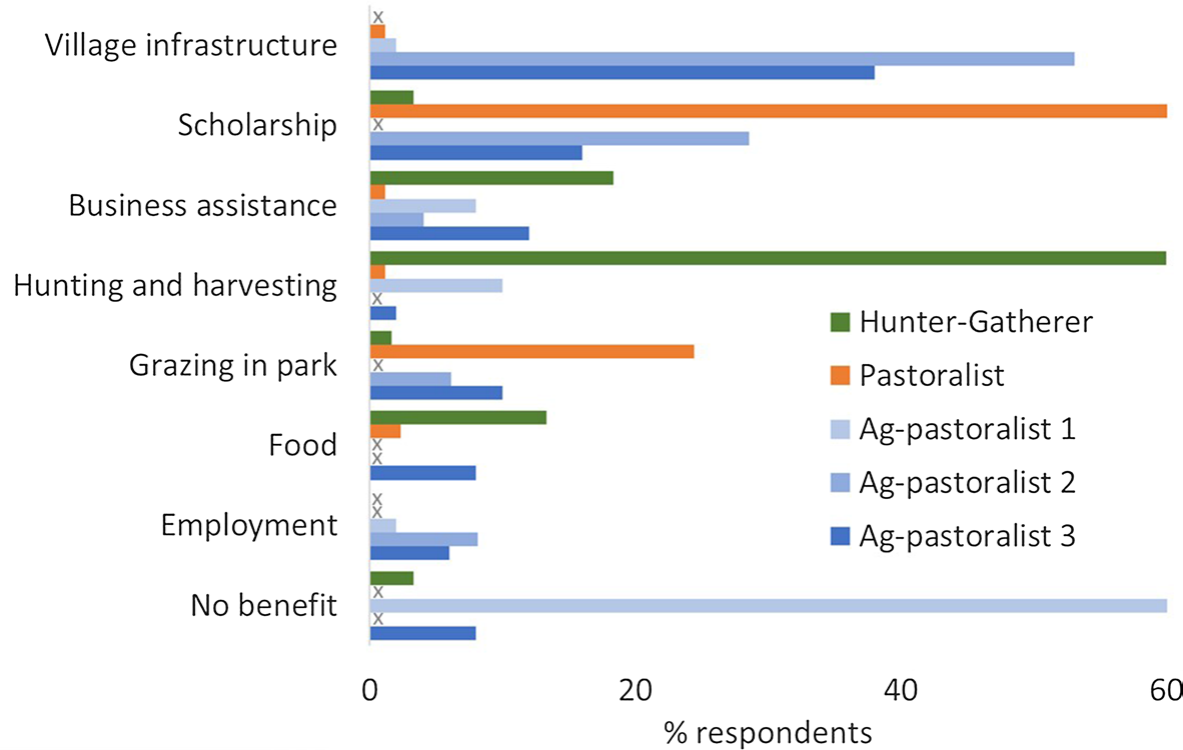
Percentage of respondents in each of five communities in northern Tanzania nominating benefits from nearby protected areas of greatest benefit to them. “x” indicates that the benefit was not nominated by the community

The benefits most appreciated by respondents varied greatly between communities (Fig. 3). Three villages rated scholarships highly—the Pastoralist village (70%), Agro-pastoralist 2 (29%) and Agro-pastoralist 3 (16%) with the latter two also giving high scores (38% and 53% respectively) to infrastructure (schools, health centres, offices). Many Pastoralist respondents (24%) listed grazing as the benefit they appreciated most. The Hunter-gatherer community rated their access to the protected areas as being the most important benefit, including their rights to hunt (60%), assistance with tourism businesses (18%) and provision of supplementary food (13%). Only 18% of respondents from Agro-pastoralist 1 village nominated any benefit as being important. Of those that did, a bee-keeping project was mentioned most frequently (Fig. 3).

### Effectiveness of conservation-related benefits in securing community support

**Fig. 4:**
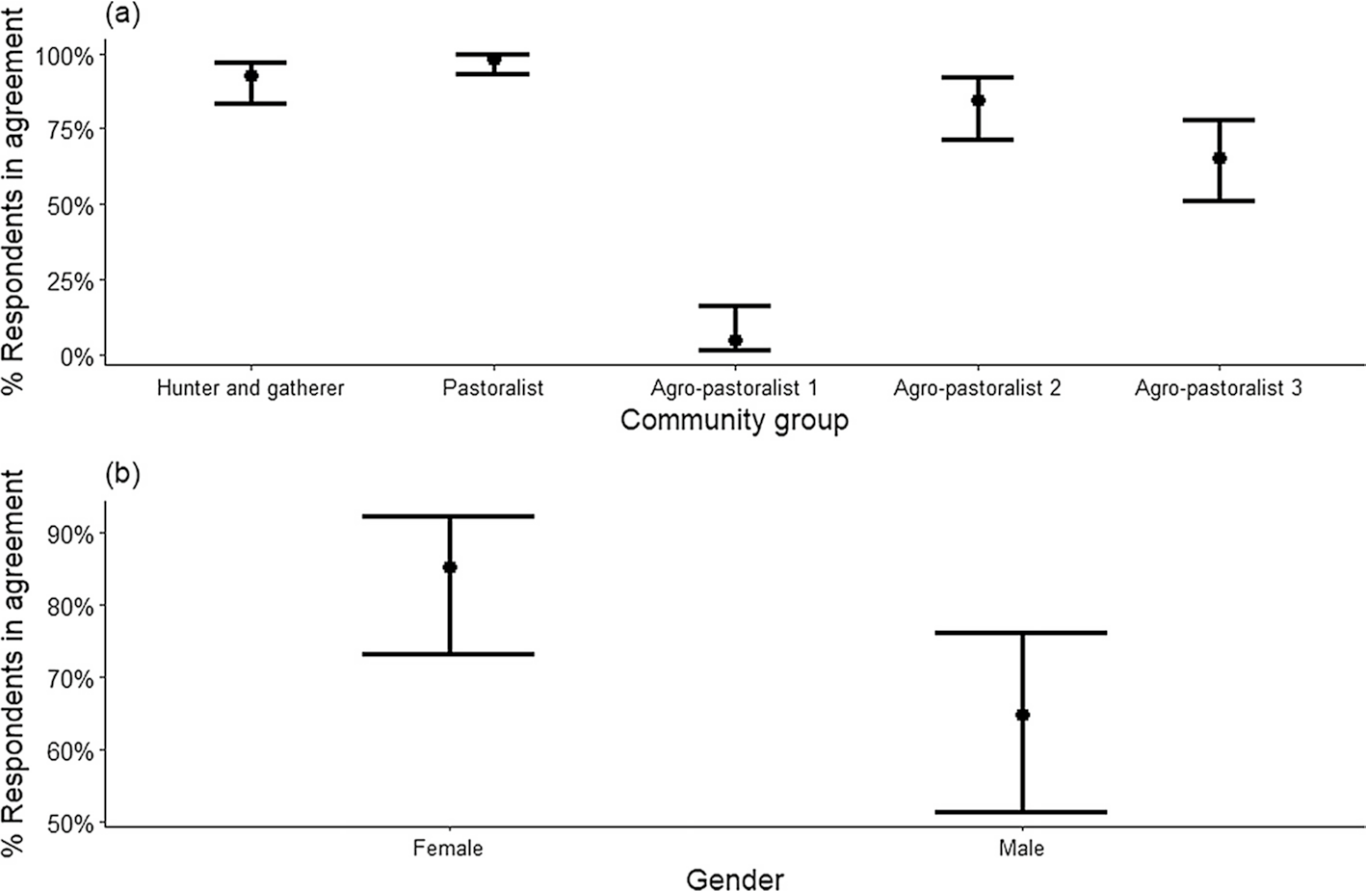
Estimated percentage of respondents in northern Tanzania agreed that benefits received encouraged them to support the conservation of nearby protected areas. This is based on a binomial multivariable model with predictors – community groups and gender. **a** shows the estimated percentage of agreement for each community group averaged across gender and **b** for gender averaged across community groups. The error bars are 95% confidence intervals (for details see Appendix 4)

Most respondents (72%) felt encouraged to support protected area conservation goals by the benefits they received (Appendix 4) but support varied significantly among villages and between genders (P < 0.001). Age and education levels were not associated with any of the agreements (P > 0.05). The most supportive were pastoralist respondents (98%), and the least supportive were the Agro-pastoralist 1 respondents (only 5%) (Fig. 4a). Across communities, women were about 20% more likely than men to agree that the benefits received encouraged them to support nearby protected areas (women: 85%, men: 65%, P < 0.001; Fig. 4b).

**Fig. 5:**
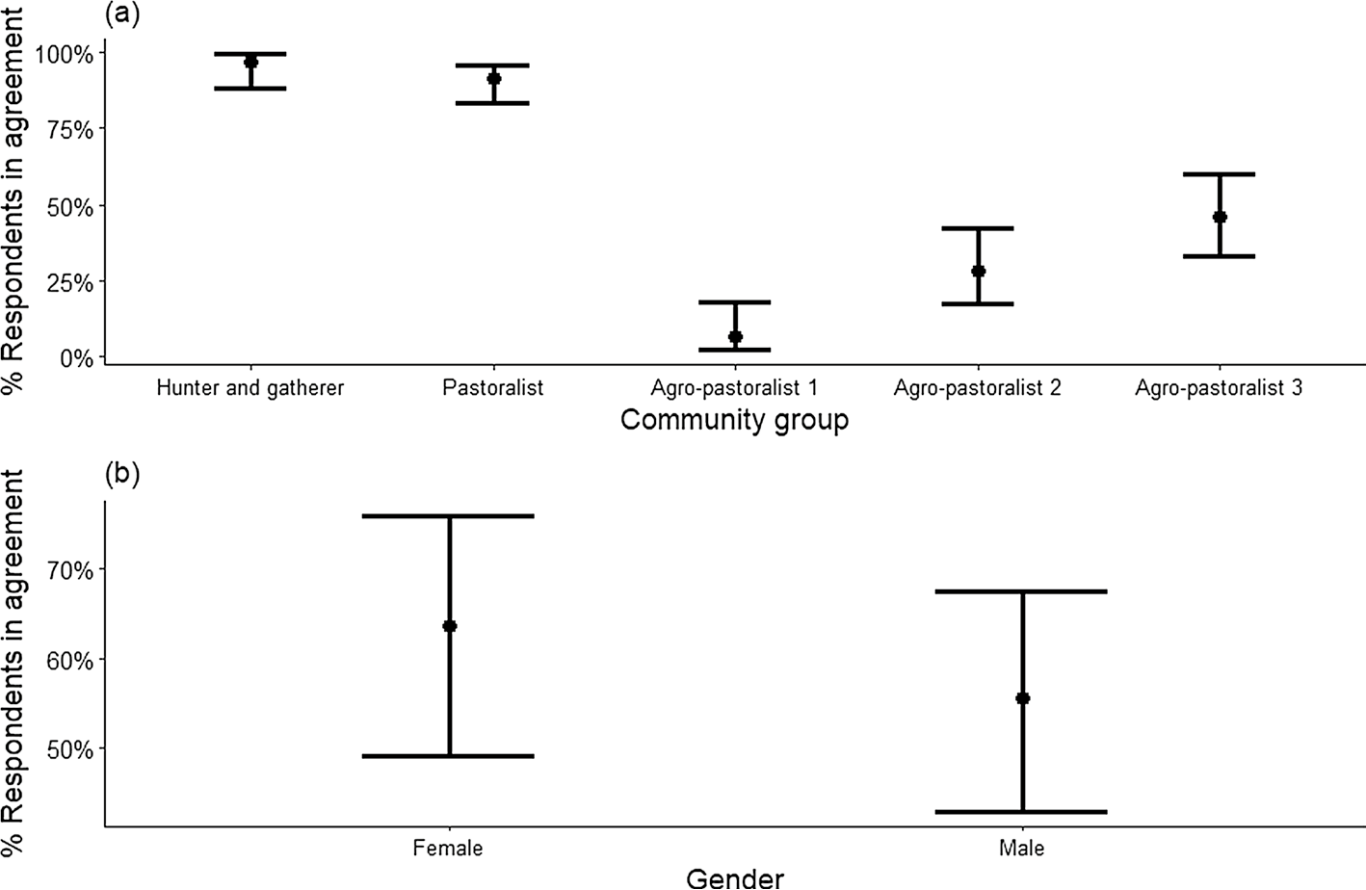
Estimated percentage of respondents in northern Tanzania agreeing that benefits from nearby protected areas helps reduce illegal activities in nearby protected areas. This is based on a binomial multivariable model with predictors – community groups and gender. **a** shows the estimated percentage of agreement for each community group averaged across gender and **b** for gender averaged across community groups. The error bars are 95% confidence intervals (for details see Appendix 2).

A similar but less pronounced pattern was apparent in the responses to the question of whether the benefits received are helpful in reducing illegal activities in the nearby protected area. In this case, it was the Hunter and gatherer respondents who were in the highest agreement (97%) whereas, only a very small proportion of respondents from Agro-pastoralist 1 village agreed (6%, P < 0.001; Fig. 5a). However, the agreement from the other Agro-pastoralist villages was also low. The difference between the responses from women (64%) and men (56%) was also smaller (Fig. 5b).

**Fig. 6:**
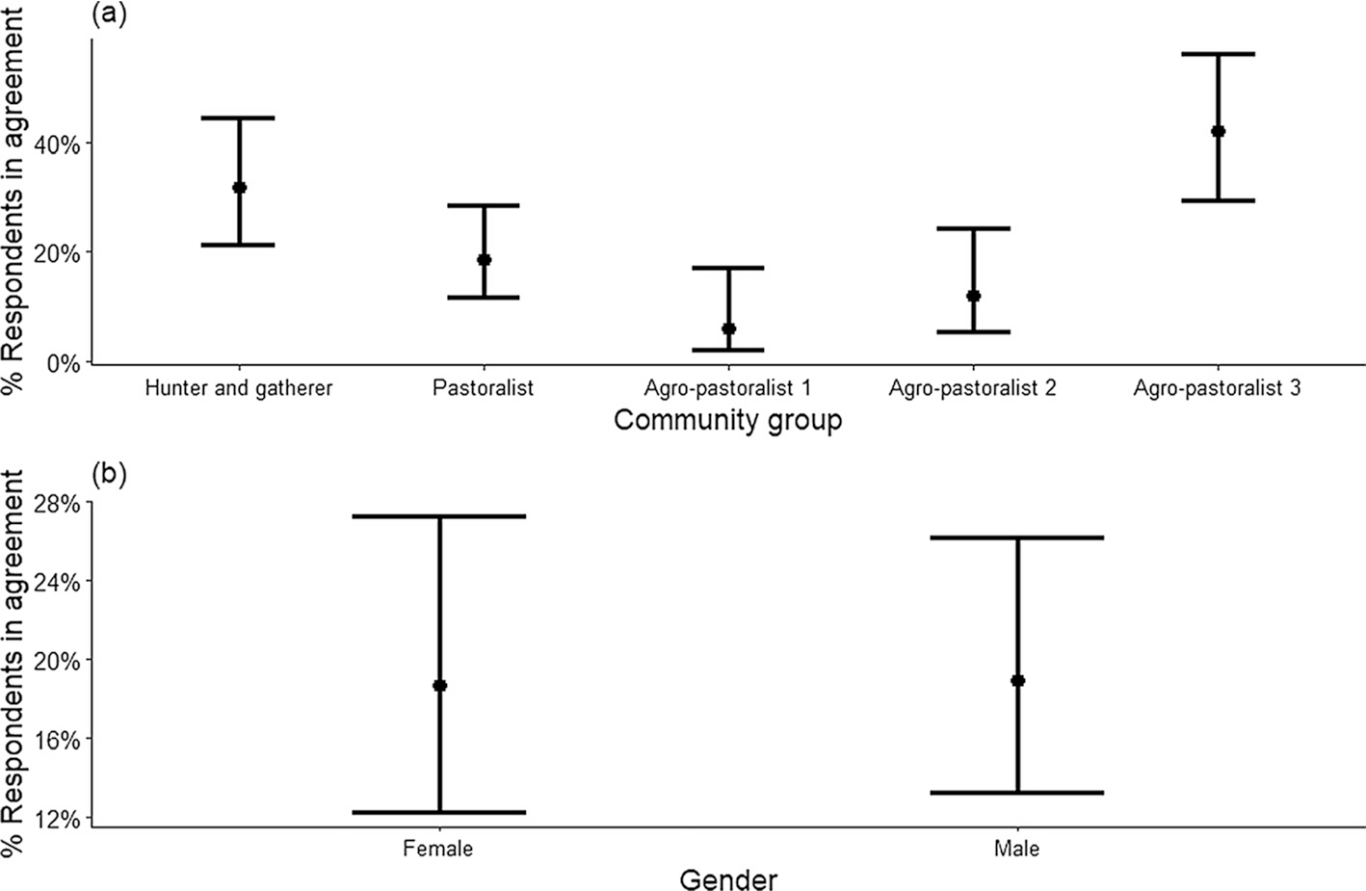
Estimated percentage of respondents in northern Tanzania agreeing that they are willing to support the existence of protected areas nearby without receiving any benefit. This is based on a binomial multivariable model with predictors – community groups and gender **a** shows the estimated percentage of agreement for each community group averaged across gender and **b** for gender averaged across community group. The error bars are 95% confidence intervals (for details see Appendix 3).

Only 22% of respondents were willing to support the existence of a protected area near their village without receiving any benefit. Respondents from Agro-pastoralist 1 village were the least likely to be supportive (6%; Fig. 6a), and only 11% of those in Agro-pastoralist 2 village and 19% of pastoralists were supportive. Support was highest among the Hunter and gatherer (32% respondents) and in Agro-pastoralist village 3 (42% respondents). However, in Agro-pastoralist village 3, it was unclear whether respondents considered the benefits derived from investment in tourism on their land as a benefit that ultimately derived from their proximity to a protected area. The percentage of women agreeing (18.7%) was almost identical to that of men (18.9%; Fig. 6b).

## Discussion

Ensuring the suitability of benefits delivered by conservation institutions to local communities whose rights and access to resources they compromise is a global issue that requires urgent attention for better conservation outcomes (Veldhuis et al. 2019; Dawson et al. 2021). This study is the first to report on the suitability of benefit-sharing mechanisms in the GSE region. We found that all benefits provided by conservation institutions operating in the GSE fell into the categories of social services provision, livelihood support and employment. However, the types of benefit within these categories varied significantly among wildlife institutions in terms of magnitude and frequency. In addition, the level of acceptance varied greatly among communities. Some respondents expressed concern that the benefits did not compensate for the high costs incurred from living with wildlife but scholarships for students were nevertheless particularly well received across all communities. Importantly, most respondents also considered that the benefits they received from conservation institutions were effective in encouraging them to support protected area goals. In fact, most respondents indicated that without some benefit sharing they would not support conservation. This suggests that local people are willing to support conservation outcomes but require conservation institutions to consider the costs incurred by communities, their livelihood needs and access to natural resources or other benefits. This community viewpoint is yet to be properly understood and acted upon when planning for conservation benefit-sharing mechanisms (Bennett et al. 2019; Dawson et al. 2021). We discuss below in detail the variety of benefits provided, local perceptions, and the effectiveness of benefits in securing support for conservation goals. This understanding will inform future planning of benefit-sharing mechanisms for better conservation outcomes in the GSE and elsewhere.

### Variety in the types of benefit received by the communities in GSE

The variation in the types of benefit received by the communities from conservation institutions in GSE might be explained by four major reasons. First, this is influenced by differing institutional policies and legislation governing protected area categories, which define the relationship and interaction between communities and the protected areas (Mariki 2013; Kegamba et al. 2022). For example, while the communities living inside Ngorongoro Conservation Area cannot practice agriculture (NCAA 2002), the NCAA management has specific responsibility for safeguarding and promoting the interest of Indigenous people living within the protected area’s borders (NCAA 2002). The NCAA has addressed this potential policy conflict by supplying food grains to reduce food scarcity and insecurity in the communities (URT 2022b). Before these initiatives, there was great concern about a significant deficit in Ngorongoro residents’ dietary needs (Boone et al. 2006; Galvin et al. 2006). In addition, due to other limitations, including the lack of public transport in the Ngorongoro Conservation Area, the management provided an ambulance for any health emergency to residents. Other conservation institutions in the GSE do not offer this type of benefit, possibly because they are not bound to do so under their legislation. Another difference is the compensation paid by the Ministry of Natural Resources and Tourism for losses to dangerous wildlife (elephants, lions, buffalo, hyenas, hippopotamus, rhinoceros and crocodiles; URT 2011). As per policy, compensation should cover losses of crops and stock within village boundaries, except inside Ngorongoro Conservation Area. However, since 2014 payments have been constrained to death or injury of people. This is because too many claims for stock losses were considered fraudulent (Head of Community Development Department; personal communication 2022). This situation potentially reflects issues with the framing or implementation of policy.

Secondly, the variation in the types of benefit received is explained by a lack of transparency/uniformity in allocating the benefits. For example, Serengeti National Park under TANAPA provides benefits through the ‘Support for Community Initiated Projects’ (SCIP), which has a set of criteria and priorities (Vedeld et al. 2012; Mariki 2013). According to the Serengeti National Park Acting Head of Community Outreach Department (personal communication 2022), the Department receives applications from the villages within 10 km from the park boundary. A technical committee from SENAPA appointed by the Department evaluates and prioritises the applications, within a defined budget. One of the primary evaluation criteria is that the village applying for benefits should have low levels of community poaching. However, the technical committee does not include any community representation, nor are the communities involved in setting those criteria. This lack of transparency is also apparent in other National Parks in Tanzania (Mariki 2013). For example, in Mikumi National Park, the same practices were found, and influenced by external politics in the selection and implementation of community development projects (Vedeld et al. 2012). In contrast, revenue accrued by the Ikona WMA is divided equally among all participating villages (Robinson and Makupa 2015; Kicheleri et al. 2018). Similarly, the benefit distribution procedure of the NCAA is different than most other NPs in Tanzania because the NCAA provides a package of benefits approved by the community representatives (Ngorongoro Pastoral Council) from all the villages within Ngorongoro Conservation Areas (URT 2022b). However, because the Ngorongoro Pastoral Council represents only the Maasai and Datoga pastoralists, the Hunter and gatherer people (Hadzabe) are not represented and reported that they receive fewer benefits than the Datoga or Maasai people living in Ngorongoro.

Thirdly, differences in the type of benefit received might be due to the number of protected areas surrounding a village. Multiple conservation institutions border some villages such as Agro-pastoralist 2 and Agro-pastoralist 3 (Fig. 1). This means benefits may be received from two institutions by the same village. In addition, some of the non-governmental organizations or tourism companies operating inside the protected area offer benefits to the same privileged villages located close to their area of operations. Therefore, these villages are able to draw on a portfolio of benefits from different conservation institutions in the area (Gardali et al. 2021). In contrast, a community like the highly dissatisfied Agro-pastoralist 1 village, which bordered a single conservation institution, was only eligible to receive benefits from a single source.

Location of a community in relation to park’s administrative offices also matters. In this study, the closer a community was to a park’s administrative offices, the greater the opportunity for residents to benefit. Tourists pass Agro-pastoralist 2 and Agro-pastoralist 3 communities because they are close to the administrative centre and main access point to the GSE (Fig. 1). Ezebilo and Mattsson (2010) found in the Okwangwo Division of the Cross River National Park in Nigeria that communities accessible to tourists were more likely to invest in tourism income generating activities than those further away. Close contact with the park authority may also provide opportunities for greater influence on the management to improve infrastructure for communities that are nearby (Ezebilo and Mattsson 2010). In addition, some of the non-governmental organizations or tourism companies operating inside the protected area offer benefits to the same privileged villages because they are located close to their area of operation. Our findings align with those of Makupa (2013), who found that the Agro-pastoral 3 community, which is also among the participating villages in Ikona WMA, receives more conservation benefits than any other village in the western Serengeti.

Finally, the differences in the types of benefit received might depend on the amount of revenue generated by a protected area that a village is bordering and the demand for the benefits. For example, Ngorongoro Conservation Area and Serengeti National Park are both leading conservation institutions in Tanzania for tourism revenue collection (URT 2018). However, the NCAA is obliged to support only 25 villages, all inside the protected area (URT 2022b). In contrast, Serengeti National Park is required to support 248 villages within 10 km of the park boundary (SENAPA Community Outreach Department). Therefore, communities living in Ngorongoro are likely to receive more benefits per village than those around Serengeti National Park.

### Acceptance and effectiveness of the benefits in securing community support

Most respondents viewed the benefits as effective in encouraging them to support the nearby protected area, and agreed that the benefits help reduce illegal activities in the protected area. Further, most respondents were not willing to support the existence of a protected area without benefits. Communities receiving more benefits from conservation also tend to be more aware of conservation activities, and have a more positive perception of the protected area, than communities receiving little benefit (Salafsky et al. 2001; Kideghesho et al. 2007; MacDonald 2010; Andrade and Rhodes 2012; Dewu and Røskaft 2018). Our findings from the GSE support this. Most respondents from Agro-pastoralist 1 village, which was receiving few benefits, did not feel encouraged to support the adjacent protected area. The respondents explained that the benefits were too small, inconsistently provided, and did not address the needs of individuals or households. This finding that insufficient benefits result in a lack of support for conservation reserves, because of the costs of wildlife damage, is consistent with previous research in Tanzania (DeGeorges and Reilly 2009; Vedeld et al. 2012; Mwakaje et al. 2013).

Poverty and unemployment are the key community drivers for poaching that need to be addressed for the local people living in and close to the conservation areas (Loibooki et al. 2002; Kideghesho 2016). Development projects sponsored by the Tanzanian national government at the village level include an expectation of community co-contribution. This takes the form of all able-bodied people contributing cash or labor (Kelsall and Mercer 2003). If a person fails to contribute, they are fined or their property confiscated (Kelsall and Mercer 2003). Thus, funding from conservation institutions for village development projects relieves pressure on community residents to contribute or face penalties. Similarly, some respondents explained that livelihood support projects such as beekeeping, COCOBA and entrepreneurship loans help provide employment and ultimately reduce poaching. In addition, the number of young people receiving scholarships to attend college helps to minimize the number of under-employed people who may engage in poaching activities.

The cost of living with wildlife influences community perceptions of the benefits received from the GSE-protected areas. In one study, the benefits received by the communities in the GSE were found to be insignificant compared to the cost incurred from wildlife (Mwakaje et al. 2013). Similarly, most respondents in this study, especially in the agro-pastoralist communities in western Serengeti, viewed the benefits received as insufficient compared to the cost incurred from wildlife. It should be noted that local people west of Serengeti have been negatively affected by human-wildlife conflicts for a long time (Kideghesho et al. 2007; Eustace et al. 2018; Snyder et al. 2021; Matata et al. 2022). The human-wildlife conflict in western Serengeti include crop raiding, mainly by elephants (*Loxodonta africana*) (Chamba 2018; Snyder et al. 2021; Matata et al. 2022), livestock depredation by carnivores such as spotted hyena (*Crocuta crocuta*), lion (*Panthera leo*), and leopard (*Panthera pardus*) (Holmern et al. 2007; Røskaft et al. 2013), and also attacks on humans by elephants (Mwakatobe et al. 2020). The communities experiencing more costs or losses from human-wildlife conflicts in western Serengeti had a more negative attitude towards the protected areas than communities experiencing less cost (Kideghesho et al. 2007). The compensation available for losses from some particular wildlife species (URT 2011) is unlikely to be sufficient. Similar findings have been reported in studies from elsewhere in Sub-Saharan Africa (Akama et al. 1995; Snyman 2014; Dewu and Røskaft 2018). For example, the communities suffering more loss to wildlife were less likely to perceive the conservation benefits positively from the Hwange National Park in Zimbabwe (Parker et al. 2022).

The conflict between the protected areas’ management or government and the community also influences communities’ perspectives toward the benefits received from protected areas in the GSE. For example, some communities have a strong fear of eviction from Ngorongoro Conservation Area. The same concern was reported by Melubo and Lovelock (2019) for the Maasai communities living in the same protected area. During the data collection of this study, there were ongoing protests by the Maasai people living in the Ngorongoro Conservation Area against a planned relocation by the Tanzanian government (Mittal 2022)—relocation now confirmed as legal by the East African Court of Justice (Sutherland 2022). A similar conflict is ongoing for the Maasai people living in Loliondo Game Controlled Area (IWGIA 2022). They were deeply concerned that they might miss out on benefits in places to which they may be relocated. In addition, the Hadzabe people described how their land is being invaded by other communities, especially Mbulu farmers and Datoga herders and farmers with no active action by the local government to stop this invasion (Mahiya et al. 1999; Madsen 2000; Levi and Durham 2015).

Another point of conflict was the location of the boundary of a protected area. Respondents from two communities in our study claimed that the boundary of Ikorongo Game Reserve was wrongly placed by the game reserve authority under the use of force. A similar claim has been recently reported by Matata et al. (2022). The communities expressed those concerns as being among the reasons for not accepting the benefits received because the protected area authorities have taken their land which could have been used for cultivation and livestock grazing. The location of the boundary is particularly important for compensation. Losses of crops to wildlife can be claimed only if they are within the boundaries of a village and consistent with the village land use plan. Losses receive no compensation if within a 0.5 km buffer (URT 2011).

The penalties given to wildlife offenders by the court under the Wildlife Conservation Act No. 5 of 2009, perceived to be substantial, can be another source of conflict between protected areas and nearby communities. Penalties include confiscation of livestock found illegally in the protected areas, a minimum sentence of 20 years in prison for possession of bushmeat, even though bushmeat hunting is an important livelihood strategy for people in the western Serengeti (Kideghesho et al. 2005; Mwakaje et al. 2013). The penalties stand in strong contrast to the compensation received from government for loss of domestic stock to wildlife, which covers only a portion of their economic and social value to their owners, can take up to three years to settle and carry their own high costs if the claim is deemed to be fraudulent. In 2020, the Tanzanian government started selling bushmeat to the public legally in 23 regions across the country at a reduced price (Issa 2020), but excluded the communities in the GSE.

### Variation of gender perspectives on the benefits received

The results indicate the differences in perspective between women and men toward the benefits received might be attributed to gender inclusion in the management of natural resources. Women have less access to information and are under-represented in natural resources decision-making than men (Ezebilo and Mattsson 2010; Ndungo et al. 2010). Most opportunities arising from conservation projects are taken by men who tend to hold most leadership positions such as village leaders and other elite community roles. It has been widely documented that gender-differentiated roles, knowledge and preferences exist in these communities (Ndungo et al. 2010; Phiri et al. 2022). In Sub-Saharan Africa, women in rural areas rely on natural resources for fuel wood, fodder, water and medicinal plants, so are more directly connected to protected areas for their immediate household needs (Mago and Gunwal 2019). However, when these resources are found on the village land and are accessible with no restrictions, women do not have to negotiate or interact with the park administration. In contrast, men are more likely to be exposed to the park administration, harassment and fines by wildlife rangers. According to (Kideghesho et al. 2007), the risks are higher for men who are found illegally grazing livestock, hunting, mining, and logging in the protected areas. Thus, men are in direct conflict with conservation, and more likely to be the subject of conservation laws. This may explain the differences in gender perceptions of the benefits received.

### Management implications

Conservation is more likely to be sustained in the long term if the local communities have the opportunity to use resources for improved livelihoods at a sustainable scale (Fyumagwa et al. 2015). The outcome of benefit sharing should be to engender support for conservation objectives. Our results suggest that one current failing of benefit provisions is that they are not guaranteed by conservation institutions. This is despite legally binding mechanisms being a key strategy for encouraging the implementation of Article 1 of the Convention on Biological Diversity for fair and equitable sharing of benefits generated from conservation (MacDonald 2010). Inclusion of benefit-sharing schemes within the institutional arrangements, such as the policies and legislation relevant to the conservation institutions in the GSE, could guarantee the availability of benefits to the communities. As a precursor to this, there is a need to standardize the benefit-sharing frameworks for transparency and take into consideration and recognize the locals’ perspectives. Co-designing mechanisms in consultation with communities is a strategy for creating transparency and addressing a lack of trust in benefit-sharing mechanisms.

To achieve this, we suggest that non-governmental organizations and tourism companies operating inside the protected area in the GSE should expand their support to communities that have not been privileged by their geography, such as being near a park gate, but still suffer the negative consequences of adjacency. There are also opportunities to promote alternative benefit-sharing models. One that has been successful among the respondents was a business model of benefit-sharing (Gorman and Ennis 2022) adopted by villagers in the Agro-pastoral 3 group who engaged in conservation business with foreign and local tourism investors on their land. Another successful example is Communal Conservancies in Namibia (Naidoo et al. 2016). However, this must be done with care—a similar community joint venture partnership in the Loliondo Game Controlled Area in the eastern GSE was unsuccessful because of centralised ownership and control of wildlife on community land by the Tanzanian Government (Gardner 2012). We propose that a community-based enterprise model should be supported and promoted to other communities as the businesses provide direct economic benefits to the communities.

A more equitable distribution of benefits to the communities bordering the protected areas in the GSE is essential to reducing disparity among local people and gathering more support for conservation strategies. We argue that, if the Ngorongoro Conservation Area is going to exist with the current multiple land-use model, where the communities live inside the conserved area, there should be equal representation of all the communities in the council. We further suggest that, if the current bushmeat selling practice is to be retained by the Tanzanian Government, the communities living at the edges of protected areas should have bushmeat selling points like other communities in other regions of Tanzania. Furthermore, the compensation assessment process and subsequent payments should be reviewed to ensure the process is transparent and provides adequate recompense for losses. It is important to consider and resolve underlying conflicts between local communities and conservation authorities to build a good, trustworthy relationship and win community support for implementing conservation projects (Dickman 2010).

## Conclusion

Differences among communities for the types, amount, and how often the benefits are received can be influenced by factors that include differences in the conservation institutional policies and practices, the number of protected areas a community borders, proximity to the conservation institutional administrative and operational areas, and the amount of revenue generated by a protected area and the number of villages it supports. The acceptance of benefits was highly variable and influenced by the scale of the benefits received compared to the perceived cost of living with wildlife and the conflict between the management of protected areas and the communities. While most respondents viewed the benefits as effective in encouraging them to support the goals of the nearby protected areas, they would be unwilling to provide support without benefits. Exceptions were the Hunter and gatherer and Agro-pastoralist 3 communities who rely less on the direct benefits provided by the protected area’s authorities, and instead benefit directly from the existence of those protected areas from tourism. Tourists visit some Hunter and gatherer camps within or near protected areas and provide those communities with revenue. Thus, these camps inside or near protected areas have better access to resources, compared to those further away from tourist activities. Those living outside protected areas also have to deal with their land being invaded by other communities. An Agro-pastoralist 3 villager that has invested in tourism operating companies also receives direct benefits. Conservation institutions need to understand the actual (tangible and intangible) costs that the local communities experience from living with wildlife. Accordingly, institutional policies need to be investigated to identify where they serve as a barrier to improving the benefit-sharing mechanisms in the GSE. For example, business opportunities for local people need to be expanded, and barriers to local business development understood and minimized. Involvement of both genders in conservation decision making is also vital in gaining overall community support for conservation. We recommend that future research should focus on conservation-related local community demands and aspirations, and on developing co-designed benefit-sharing mechanisms to deliver effective conservation outcomes as well as enhance community livelihoods. In addition, the impact of wildlife on the local people’s livelihood strategies in the GSE should be thoroughly investigated to find solutions for delivering effective conservation programs.

## Supplementary Information

Below is the link to the electronic supplementary material.

Supplementary material 1 (DOCX 14.6 kb)

Supplementary material 2 (DOCX 14.6 kb)

Supplementary material 3 (DOCX 14.7 kb)

Supplementary material 4 (DOCX 38.8 kb)

## Data Availability

Data from this study comprises notes taken during focus group discussion and bespoke forms completed during household survey. Data have been de-identified so that participants remain anonymous. These data may be provided subject to ethics approval.
