# Supplementary material for: Conservation benefit-sharing mechanisms and their effectiveness in the Greater Serengeti Ecosystem: local communities’ perspectives
Source: Biodivers Conserv. 2023 Apr 6;32(6):1901–30. doi: 10.1007/s10531-023-02583-1 (PMC10077326; doi:10.1007/s10531-023-02583-1)
Supplement: Supplementary file 1 — Supplementary material 1 (DOCX 14.6 kb) [file 10531_2023_2583_MOESM1_ESM.docx]

**Appendix 1**. Respondent’s probability of agreement that benefits received encouraged them to support nearby protected areas. These probabilities are based on a binomial multivariable model with community group and gender as predictors. The probabilities shown are averaged across community groups and gender

| **Community groups** | **Average Probability (%)** | **95% CI (%)** | |  |  |
| --- | --- | --- | --- | --- | --- |
|  |  | **Asymp.LCL** | **Asymp.UCL** |  |  |
| Agro-pastoralist 1 | 4.5 | 1.1 | 16.3 |  |  |
| Agro-pastoralist 2 | 84.4 | 71.6 | 92.0 |  |  |
| Agro-pastoralist 3 | 65.6 | 51.0 | 77.7 |  |  |
| Hunter and gatherer | 92.9 | 83.4 | 97.1 |  |  |
| Pastoralist | 98.2 | 93.0 | 99.6 |  |  |
| **Gender** |  |  |  |  |  |
| Female | 85.1 | 73.0 | 92.3 |  |  |
| Male | 64.6 | 51.3 | 76.0 |  |  |
