# Supplementary material for: Conservation benefit-sharing mechanisms and their effectiveness in the Greater Serengeti Ecosystem: local communities’ perspectives
Source: Biodivers Conserv. 2023 Apr 6;32(6):1901–30. doi: 10.1007/s10531-023-02583-1 (PMC10077326; doi:10.1007/s10531-023-02583-1)
Supplement: Supplementary file 2 — Supplementary material 2 (DOCX 14.6 kb) [file 10531_2023_2583_MOESM2_ESM.docx]

**Appendix 2**. Respondent’s probability of agreement that benefits received are helpful in reducing illegal activities in the nearby protected area. These probabilities are based on a binomial multivariable model with community group and gender as predictors. The probabilities shown are averaged across community groups and gender.

| **Community group** | **Average Probability (%)** | **95% CI (%)** | |
| --- | --- | --- | --- |
|  |  | **Asymp.LCL** | **Asymp.UCL** |
| Agro-pastoralist 1 | 6.4 | 2.1 | 18.0 |
| Agro-pastoralist 2 | 28.3 | 17.5 | 42.3 |
| Agro-pastoralist 3 | 46.1 | 32.9 | 6.0 |
| Hunter and gatherer | 96.7 | 87.9 | 99.2 |
| Pastoralist | 91.2 | 83.1 | 95.6 |
| **Gender** |  |  |  |
| Female | 63.6 | 49.1 | 76.0 |
| Male | 55.5 | 42.9 | 67.5 |
