# Supplementary material for: Conservation benefit-sharing mechanisms and their effectiveness in the Greater Serengeti Ecosystem: local communities’ perspectives
Source: Biodivers Conserv. 2023 Apr 6;32(6):1901–30. doi: 10.1007/s10531-023-02583-1 (PMC10077326; doi:10.1007/s10531-023-02583-1)
Supplement: Supplementary file 3 — Supplementary material 3 (DOCX 14.7 kb) [file 10531_2023_2583_MOESM3_ESM.docx]

**Appendix 3.** Respondent’s probability of agreement that they are willing to support the existence of protected areas nearby without receiving any benefit. These probabilities are based on the binomial multivariable model with community group and gender as predictors. The probabilities shown are averaged across community groups and gender

| **Community group** | **Average Probability (%)** | **95% CI (%)** | |
| --- | --- | --- | --- |
|  |  | **Asymp.LCL** | **Asymp.UCL** |
|  |  |  |  |
| Agro-pastoralist 1 | 5.9 | 1.9 | 17.1 |
| Agro-pastoralist 2 | 11.9 | 5.5 | 24.2 |
| Agro-pastoralist 3 | 41.9 | 29.2 | 55.9 |
| Hunter and gatherer | 31.7 | 21.2 | 44.4 |
| Pastoralist | 18.6 | 11.6 | 28.3 |
| **Gender** |  |  |  |
| Female | 18.7 | 12.3 | 27.3 |
| Male | 18.9 | 13.3 | 26.2 |
