# Supplementary material for: Conservation benefit-sharing mechanisms and their effectiveness in the Greater Serengeti Ecosystem: local communities’ perspectives
Source: Biodivers Conserv. 2023 Apr 6;32(6):1901–30. doi: 10.1007/s10531-023-02583-1 (PMC10077326; doi:10.1007/s10531-023-02583-1)

**Appendix 4.** Proportion of respondents agreeing that benefits received (a) encouraged them to support nearby protected areas and (b) helped in reducing illegal activities in the protected areas.


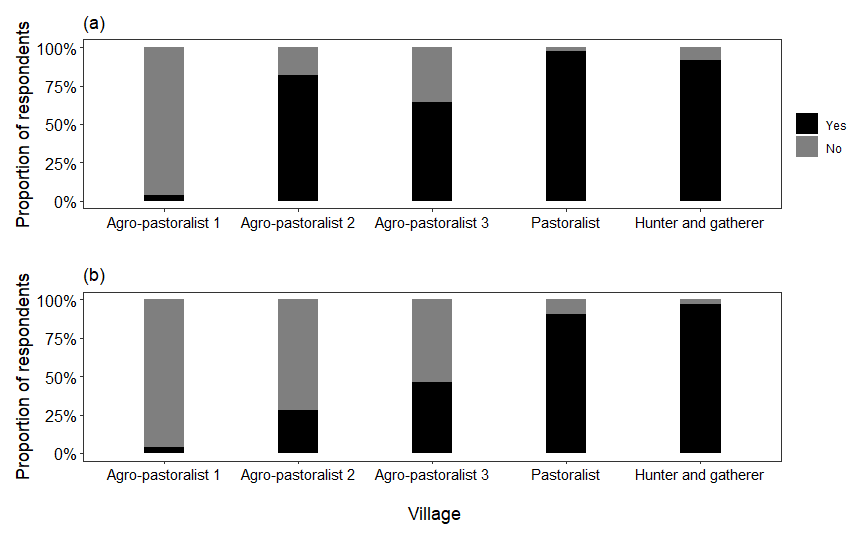

Supplement: Supplementary file 4 — Supplementary material 4 (DOCX 38.8 kb) [file 10531_2023_2583_MOESM4_ESM.docx]
